# Study on gene expression in stomach at different developmental stages of human embryos

**DOI:** 10.3389/fcell.2025.1564789

**Published:** 2025-05-30

**Authors:** Weiyu Guan, Xinran Lu, Yin Zhang, Hongping Ding, Xinmei Liu, Le Yang, Wenran Wang, Jianwu Shi, Shichun Feng

**Affiliations:** ^1^ Department of General Surgery, Nantong First People’s Hospital, Affiliated Hospital 2 of Nantong University, Nantong, Jiangsu, China; ^2^ Department of Nursing, Third People’s Hospital of Rugao, Nantong, Jiangsu, China; ^3^ Basic Medical Research Centre, Medical School, Nantong University, Nantong, Jiangsu, China

**Keywords:** stomachs, human embryos, transcriptomic sequencing, development, gene expression

## Abstract

**Background:**

The proper development of embryonic stomach in human is essential for the functionality of the adult stomach. However, the key genes, biological processes, and signaling pathways that influence stomach development in human embryogenesis are not yet fully understood.

**Methods:**

In this study, stomach samples were obtained from human embryos at developmental stages ranging from two to seven months. Through transcriptomic sequencing, we identified the differentially expressed genes and enrichment processes in the stomach at various developmental phases.

**Results:**

The results of this study indicate that genes associated with embryonic organ morphogenesis, digestive tract development, and gastric acid secretion displayed elevated expression during the early developmental stages. Additionally, a number of genes linked to cilium assembly and organization, peptide and hormone secretion and transportation, and immune response, showed increased expression during stomach maturation. Our findings elucidate that both the morphological and functional aspects of the stomach develop during the early stages of embryonic development. As gastric development, the stomach progressively acquires additional functions. This research provides insights into the intricate regulatory networks among the genes involved in embryonic digestive tract development, digestion and embryonic organ morphogenesis. Therefore, the formation of human embryonic stomach necessitates the synergistic regulation of a plethora of genes. Notably, this study not only identified traditionally recognized genes but also revealed many previously uncharacterized genes that play potential roles in stomach development and its functions.

**Conclusion:**

These findings establish a crucial basis for future studies on stomach development and the disorders arising from fetal stomach abnormalities.

## Introduction

The stomach plays a pivotal role in the human digestive system. The stomach is an evolutionarily diverse organ that performs multiple functions, including food digestion, immune defense, and the hormonal regulation of metabolic homeostasis. Vertebrates have developed variations in the structural and histological organization of the stomach in response to specific dietary needs and habits (Kim and Shivdasani, 2016). In human, the gastric mucosa consists of glandular, columnar epithelium and is regionally differentiated into two distinct compartments. These compartments contain specialized cell types that function complementarily. The proximal region of the stomach encompasses fundic glands, which comprise acid-secreting parietal cells, enzyme-producing chief cells, and cells that generate protective mucus. In contrast, the distal region of stomach, known as the antrum, primarily contains mucous cells and endocrine cells including G-cells responsible for gastrin secretion (McCracken and Wells, 2017). In addition, mice possess an expansive forestomach situated anteriorly, lined with a stratified squamous epithelium akin to that found in the esophagus (Kim and Shivdasani, 2016).

When a segment of the yolk sac is incorporated into the embryo, it results in the formation of the primitive gut. This occurs as a consequence of the cephalocaudal and lateral folding processes of the embryonic development. The primitive gut develops into a blind-ended tube at both the cephalic and caudal ends of the embryo, consequently giving rise to the foregut and hindgut, respectively. The central section gives rise to the midgut, which is temporarily connected to the yolk sac through the vitelline duct, also known as the yolk stalk. The foregut gives rise to the esophagus, trachea, lungs, liver, pancreas, hepatobiliary system, and stomach, while the midgut and hindgut develop into the small and large intestines, respectively (Kim and Shivdasani, 2016). Thus, the stomach is an organ that originates from the foregut. The embryonic development of the stomach can be subdivided into distinct stages. Stage 1 (4 weeks embryo): Neuroectodermal cells differentiate into the neural crest, which in turn gives rise to the primitive foregut. Stage 2 (4–5 weeks embryo): The foregut subdivides into three regions: the forestomach, the duodenum, and the ileum. Stage 3 (5–6 weeks embryo): The anterior portion of the stomach undergoes gradual expansion to constitute the initial anterior and posterior stomach. Stage 4 (6–7 weeks embryo): The anterior portion of the stomach is subdivided into two regions: the left becomes the body of the stomach, while the right develops into the fundus. Stage 5 (7–9 weeks embryo): The cardia is formed at the juncture of the anterior and posterior regions of the stomach. Stage 6 (9–12 weeks embryo): The glands and main artery of stomach fundus are formed, and the stomach body progressively matures.

Numerous signaling pathways and transcription factors have been identified as key regulators of stomach development. Retinoic acid (RA) signaling plays a critical role in foregut organogenesis and the maintenance of the foregut-midgut boundary (Molotkov et al., 2005; Wang et al., 2006). The fibroblast growth factor (FGF) signaling pathways induce posterior endoderm markers in a concentration-dependent manner while promoting the expression of midgut genes at the expense of those associated with the foregut (Wells and Melton, 2000; Dessimoz et al., 2006). Similarly, canonical WNT signaling is crucial for hindgut development, posteriorizing the foregut as a result of its activity (Gregorieff et al., 2004; McLin et al., 2007; Sherwood et al., 2011). Within the visceral endoderm, HHEX is necessary for normal primitive streak morphogenesis and is critical to appropriate foregut development (Dufort et al., 1998; Martinez Barbera et al., 2000). The transcription factors SOX2 and CDX2 demarcate the precise boundaries of the future stomach and intestine, potentially via mutual cross-antagonism (Silberg et al., 2002; Que et al., 2009; Raghoebir et al., 2012). The zinc-finger transcription factor GATA4 exhibits high expression levels in the developing glandular stomach and other gut epithelia. In chimeric mice, *Gata4*-null epithelial cells do not contribute to this tissue (Jacobsen et al., 2002), suggesting a role in the specification of stomach mucosa. BARX1 is specifically expressed in mid-gestation stomach mesenchyme. Within the adjacent stomach epithelium, BARX1 induces the secretion of WNT antagonists (sFRPs) to attenuate WNT signaling, a pathway that typically enhances intestinal development (Kim et al., 2005; Kim et al., 2007). The homeobox gene HOXA5 is prominently expressed in the mesenchyme of hindstomach and is essential for its appropriate development (Aubin et al., 2002).

In this research, we collected human embryonic stomachs from various developmental stages, including 2-month-old, 3-month-old, 4-month-old, 5-month-old, 6-month-old and 7-old month. By transcriptome sequencing, we identified genes with high expression levels and associated enrichment processes critical for the development and functioning of the stomach. Elucidating the processes and regulatory mechanisms underlying gastric embryonic development is crucial for improving the diagnosis and treatment of related congenital gastric diseases. Therefore, this study lays the foundation for researches into gastric development, tissue regeneration and gastric disease treatment.

## Materials and methods

### Collection of human embryonic stomachs

This study was approved by the Institutional Review Committees of Rugao Third People’s Hospital and the Affiliated Hospital of Nantong University (Approval No. 2020-K013). Before commencing the study, informed consent was obtained from the parents of the participants. The study utilized embryonic stomach tissues from legally aborted human fetuses ranging from 2-month-old to 7-month-old. Subsequently, these tissues were collected and preserved at −80°C. One biological stomach sample was collected at each timepoint for analysis.

### Isolation of stomach RNA

For RNA extraction, 50 mg of stomach tissues were collected from developmental stages spanning 2–7 months old. Total RNA was extracted with TRIzol reagent (Thermo Fisher Scientific, Waltham, MA, United States). The quantity and quality of RNA was measured using the Agilent 2,100 BioAnalyzer system (Agilent Technologies, Santa Clara, CA, United States) with the RNA 6000 Nanochip. The 260 nm/280 nm absorbance ratio was established as the quality control criterion, with acceptable values ranging from 1.8 to 2.2. RNA Integrity values (RIN) were 10.0 of 2-month-old, 7.3 of 3-month-old, 7.9 of 4-month-old, 6.1 of 5-month-old, 7.1 of 6-month-old, 6.0 of 7-month-old used in the analysis.

### RNA sequencing and data analysis

The RNA samples isolated from gastric tissues underwent adapter ligation at both 5′and 3′ends using T4 RNA ligase, followed by reverse transcription with adapter-specific primers to generate cDNA. The resulting cDNA libraries were subsequently amplified through PCR. The RNA libraries were purified via agarose gel electrophoresis and were further assessed. Then, sequencing was performed on the BGISEQ-500/MGISEQ-2000 system (BGI-Shenzhen, China). The raw sequencing data were quality-filtered using SOAPnuke pipeline (https://github.com/BGI-flexlab/SOAPnuke), and the resulting clean reads were archived in FASTQ format for downstream analysis. The clean sequencing reads were aligned to the human reference genome GRCh38 using HISAT (http://www.ccb.jhu.edu/software/hisat). The sequence alignment map (SAM) files generated from aligned RNA-Seq data were converted to binary alignment map (BAM) format using Samtool processing (https://github.com/samtools/samtools). The read-count matrix corresponding to each gene was systematically generated using featureCounts (https://github.com/gih0004/RNA_Seq_featurecounts).

Differential gene expression analysis was performed using the edgeR package. The statistical significance threshold was established at p < 0.05, with differential expression defined by an absolute log2 fold change (|log2FC|) > 1. Genes exhibiting a two-fold or greater change in expression levels (either upregulation or downregulation) were classified as differentially expressed genes (DEGs). To investigate the underlying biological mechanisms and pathways associated with the differentially expressed genes (DEGs), we conducted Gene Ontology (GO) functional enrichment analysis and Kyoto Encyclopedia of Genes and Genomes (KEGG) pathway enrichment analysis using the clusterProfiler package. These analyses were performed with a significance threshold of p-value  <  0.05, adjusted for multiple comparisons using the Benjamini–Hochberg false discovery rate correction method. The gene interaction networks implicated in stomach development, morphogenesis, and cell differentiation were analyzed and visualized using Cytoscape (Shannon et al., 2003).

### Quantitative real-time PCR assays

Total RNA was isolated from stomach tissues and subsequently utilized for cDNA synthesis via reverse transcription kits. Total RNA was isolated from stomach tissues and was further used to synthesize cDNA using reverse transcription kits. Real-time PCR was performed with AceQ® Universal SYBR® qPCR Master Mix (Vazyme Biotech, Nanjing, China) using a LightCycler 96 Real-Time PCR System (Roche, Basel, Switzerland). Primer sequences are provided in Table 1. The relative mRNA abundances were quantified and normalized to the mean expression level of β-actin mRNA. Fold changes in mRNA expression levels were determined by the 2^−ΔΔCT^ calculation method.

**TABLE 1:** Primers for quantitative RT-PCR.

Genes	Forward primer sequence (5′ - 3′)	Reverse primer sequence (5’ - 3′)
FGF10	TCCCAGGCCCACAAAGTTAA	GCCTTTTGCGAGTTAGGAGG
GLI2	GCCGCTTCAGATGACAGATG	GTCCTGTAAGCACCAAAGGC
PCSK5	AGTTCGACATCAGCCTTGGA	ACTACAGACACTTGCCACCA
FOXF1	GCTGAGCGAGATCTACCAGT	GAGCCCTCCTCGAACATGAA
GLI3	TGGACCAAGAATCTCCGCAT	AGTGTCCCTCTGCTTGTCTC
TCF21	AGCTACATCGCCCACTTGAG	TCAGGTCACTCTCGGGTTTC
FOXF2	GCCACAATCTCTCGCTCAAC	GAGCCCTCCTCGAACATGAA
IHH	AGTTCAGGTTGCCTCTCACA	AGGGACAGACAGTGGTTAGC
SHH	AAAGACACTCGGAAAAGGCG	CCGGGGTCCTTGTTTCCTTA
SIX2	ACTACATCGAGGCGGAGAAG	GCTGCGACTCTTTTCCTTGA
HNF1B	AGGTCCGTGTCTACAACTGG	TGTTTGGAGGAGAGGAGCTG
NTN1	CCCTGGTTACTGCCTCTTGA	TTTGCTGCCTCCTCTGAAGA
TEAD2	ACACATGACCCTCACCTGTT	GGTACACAAATCTGCCGTCC
EDN1	TGTGTCTACTTCTGCCACCT	TTCACGGTCTGTTGCCTTTG
HPN	TGTGTGGCATTGTGAGTTGG	TGGCTTCGGAGTGAGTCTTT
HOXB5	TCCGCAAATATTCCCCTGGA	GGGTCTGGTAGCGGGTATAC
BMP4	TGGGCTGGAATGACTGGATT	TGGCATGGTTGGTTGAGTTG
APLNR	TCAACCCCTTCCTCTATGCC	TGCATCTGTTCTCCACCCTT
GATA4	TGTCAACTGTGGGGCTATGT	TCATCTTGTGGTAGAGGCCG
OSR1	CCTTTCCCTGGTTCCCTCAT	GGTTGGCAAAATCAAAGCGC
SI	CAATGCTCGGTGGTTTGACT	AGCTGGCTCTTGACATGGTA
NPC1L1	TGTCCCTCATCAACCTGGTC	AACACCGCACTTCCCATAGA
SOX9	GAACGCACATCAAGACGGAG	AGTTCTGGTGGTCGGTGTAG
CD36	GGCTTAATGAGACTGGGACC	TCACCACACCAACACTGAGT
SST	AGAATGATGCCCTGGAACCT	CCGGGTTTGAGTTAGCAGATC
GHRL	CCAGCAGAGAAAGGAGTCGA	TGAACCCCTGACAGCTTGAT
NPR2	AACGCCATGCACCAGAAATT	CAGTATGGACCCCTATGCGT
FABP1	TTCAAGTTCACCATCACCGC	CTCCCCTGTCATTGTCTCCA
ACTIN	TTGTTACAGGAAGTCCCTTGCC	ATGCTATCACCTCCCCTGTGTG

### Statistical analysis

Student’s t-tests and one-way analysis of variance (ANOVA) were employed to analyze differences between dual and multiple groups. All measured data were presented as mean ± standard deviation (SD). Statistical significance was established using GraphPad Prism 7.0 (GraphPad Software, Inc., San Diego, CA, United States) with a p-value less than 0.05.

## Results

### Analysis of high-expression genes and biological processes in the stomach of 3-month-old human embryo

**FIGURE 1:**
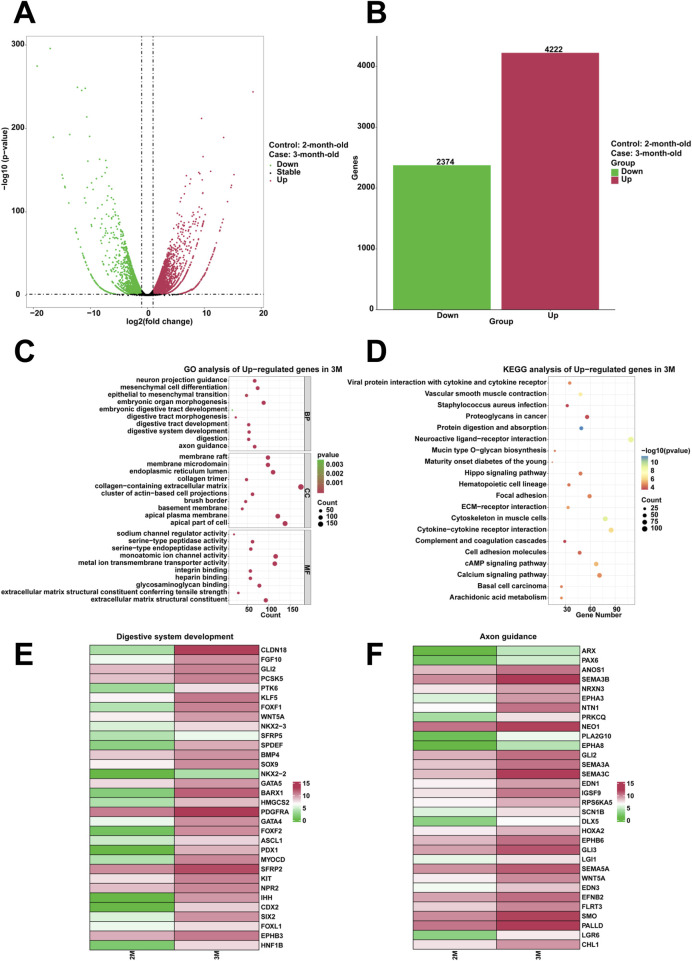
Analysis of the highly expressed genes and biological processes in the stomach of 3-month-old human embryo. **(A)** Volcano plot illustrating the differentially expressed genes (DEGs) between the 2-month-old and 3-month-old stomachs. **(B)** Statistical analysis of 4,222 upregulated and 2,374 downregulated genes in the stomach of 3-month-old embryo. **(C)** Gene Ontology (GO) analysis of the upregulated genes identified in the stomach of 3-month-old embryo. **(D)** KEGG pathway analysis of the upregulated genes identified in the stomach of 3-month-old embryo. **(E)** Heatmap revealed the expression genes related to digestive system development in the 2-month-old and 3-month-old stomachs. **(F)** Heatmap revealed the expression genes related to axon guidance in the 2-month-old and 3-month-old stomachs.

To examine the genes that are highly expressed and their corresponding biological functions in the stomach of 3-month-old human embryo, RNA sequencing analysis was conducted to identify differentially expressed genes (DEGs) between the stomachs of 2-month-old and 3-month-old embryos. A comparison revealed that 4,222 genes were upregulated and 2,374 genes were downregulated in the stomach of 3-month-old embryo (Figures 1A,B). To investigate the biological functions of differentially expressed genes (DEGs), GO analysis indicated the enrichment of processes associated with digestive tract development, embryonic organ morphogenesis, embryonic digestive tract development, digestion, digestive system development, mesenchymal cell differentiation, neuron projection guidance, digestive tract morphogenesis, epithelial to mesenchymal transition, axon guidance in the stomach of 3-month-old embryo (Figure 1C). Furthermore, KEGG analysis identified protein digestion and absorption as a critical signaling pathway among the upregulated genes (Figure 1D). When compared to the stomach of 2-month-old embryo, heatmaps demonstrated a significant increase in the expression of genes associated with digestive system development, including *CLDN18*, *FGF10*, *GLI2*, *PCSK5*, *PTK6*, *KLF5*, *FOXF1*, *WNT5A*, *NKX2-3*, *SFRP5*, *SPDEF*, *BMP4*, *SOX9*, *NKX2-2*, *GATA5*, *BARX1*, *HMGCS2*, *PDGFRA*, *GATA4*, *FOXF2*, *ASCL1*, *PDX1*, *MYOCD*, *SFRP2*, *KIT*, *NPR2*, *IHH*, *CDX2*, *SIX2*, *FOXL1*, *EPHB3* and *HNF1B* in the stomach of 3-month-old embryo (Figure 1E). High expression levels of axon guidance-associated genes, including *ARX*, *PAX6*, *ANOS1*, *SEMA3B*, *NRXN3*, *EPHA3*, *NTN1*, *PRKCQ*, *NEO1*, *PLA2G10*, *EPHA8*, *GLI2*, *SEMA3A*, *SEMA3C*, *EDN1*, *IGSF9*, *RPS6KA5*, *SCN1B*, *DLX5*, *HOXA2*, *EPHB6*, *GLI3*, *LGI1*, *SEMA5A*, *WNT5A*, *EDN3*, *EFNB2*, *FLRT3*, *SMO*, *PALLD*, *LGR6*, *CHL1*, were identified in the stomach of 3-month-old embryo (Figure 1F).

### Analysis of high-expression genes and biological processes in the stomach of 4-month-old human embryo

**FIGURE 2:**
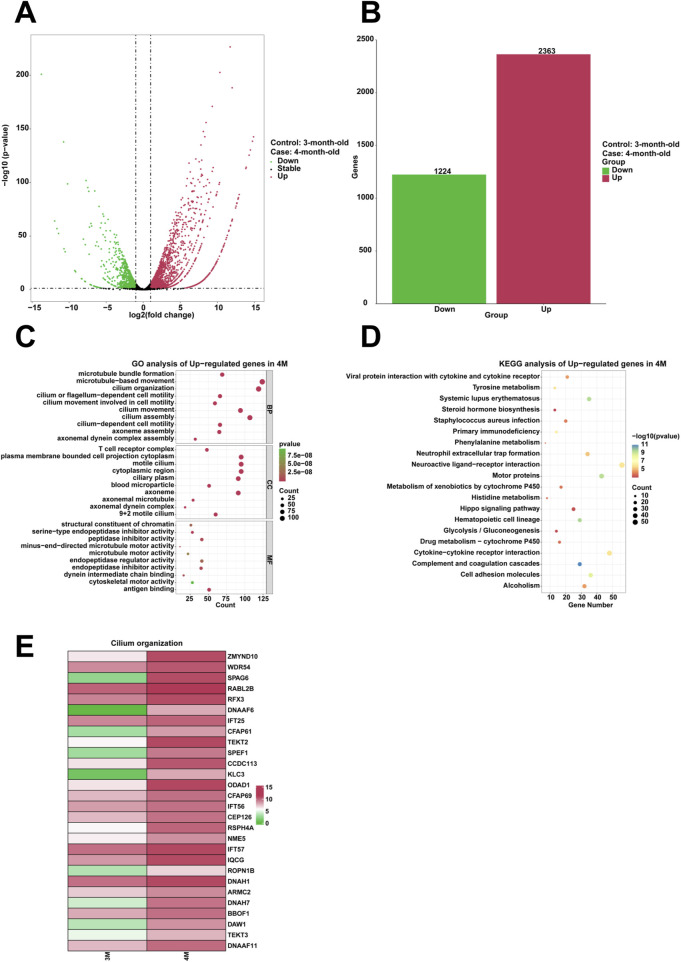
Analysis of the highly expressed genes and biological processes in the stomach of 4-month-old human embryo. **(A)** Volcano plot illustrating the differentially expressed genes (DEGs) between the 3-month-old and 4-month-old stomachs. **(B)** Statistical analysis of 2,363 upregulated and 1,224 downregulated genes in the stomach of 4-month-old embryo. **(C)** GO analysis of the upregulated genes in the stomach tissue of 4-month-old embryo. **(D)** KEGG pathway analysis of the upregulated genes identified in the stomach of 4-month-old embryo. **(E)** Heatmap revealed the expression genes related to cilium organization in the stomachs of 3-month-old and 4-month-old embryos.

To examine highly expressed genes and their associated biological functions in the stomach of 4-month-old human embryo, RNA sequencing was employed to identify differentially expressed genes (DEGs) between the stomachs of 3-month-old and 4-month-old embryos. The analysis indicated that 2,363 genes were upregulated, while 1,224 genes were downregulated in the 4-month-old stomach (Figures 2A,B). GO analysis revealed the enrichment of processes related to cilium movement, cilium organization, cilium assembly, cilium or flagellum-dependent cell motility, cilium-dependent cell motility, cilium movement involved in cell motility within the 4-month-old stomach (Figure 2C). KEGG analysis demonstrated that the complement and coagulation cascades were significantly enriched among the upregulated genes (Figure 2D). Compared to the 3-month-old stomach, heatmaps illustrated a notable increase in the expression of genes associated with cilium organization in the 4-month-old stomach, including *ZMYND10*, *WDR54*, *SPAG6*, *RABL2B*, *RFX3*, *DNAAF6*, *IFT25*, *CFAP61*, *TEKT2*, *SPEF1*, *CCDC113*, *KLC3*, *ODAD1*, *CFAP69*, *IFT56*, *CEP126*, *RSPH4A*, *NME5*, *IFT57*, *IQCG*, *ROPN1B*, *DNAH1*, *ARMC2*, *DNAH7*, *BBOF1*, *DAW1*, *TEKT3*, and *DNAAF11* (Figure 2E).

### Analysis of high-expression genes and biological processes in the stomach of 5-month-old human embryo

**FIGURE 3:**
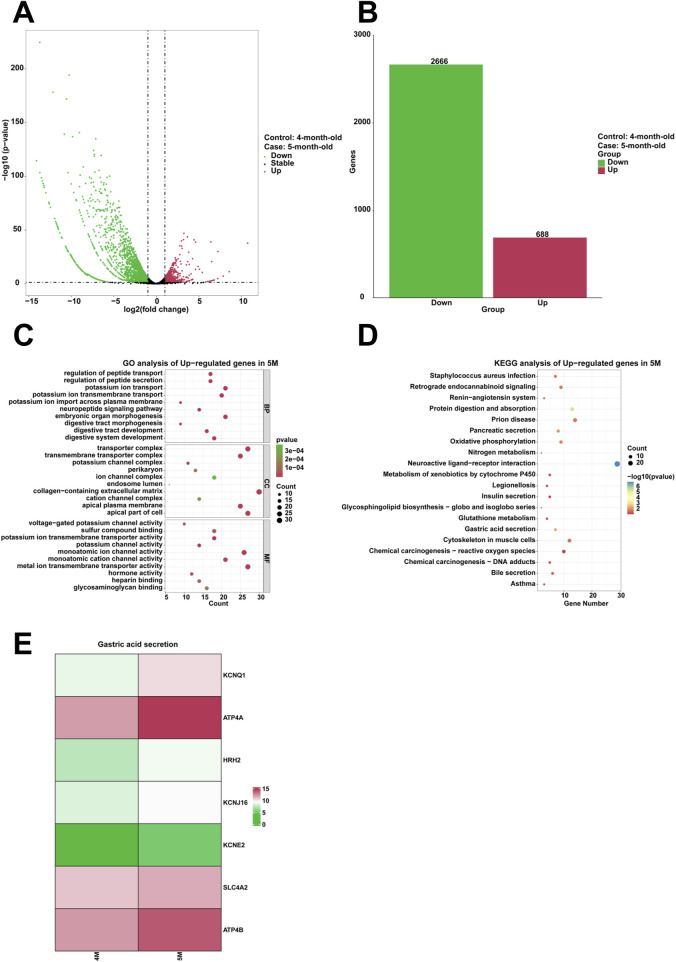
Analysis of the highly expressed genes and biological processes in the stomach of 5-month-old human embryo. **(A)** Volcano plot illustrating the differentially expressed genes (DEGs) between the stomach tissues of 4-month-old and 5-month-old embryos. **(B)** Statistical analysis of 688 upregulated and 2,666 downregulated genes in the stomach of 5-month-old embryo. **(C)** GO analysis of the upregulated genes in the stomach tissue of 5-month-old embryo. **(D)** KEGG pathway analysis of the upregulated genes identified in the stomach of 5-month-old embryo. **(E)** Heatmap revealed the expression genes related to gastric acid secretion in the 4-month-old and 5-month-old stomachs.

To analyze the genes that are highly expressed and their corresponding biological functions in the stomach of 5-month-old human embryo, RNA sequencing analysis was conducted to identify differentially expressed genes (DEGs) between the stomachs of 4-month-old and 5-month-old embryos. A comparison revealed that 688 genes were upregulated and 2,666 genes were downregulated in the stomach of 5-month-old embryo (Figures 3A,B). To investigate the biological functions of differentially expressed genes (DEGs), GO analysis indicated the enrichment of processes associated with digestive system development, potassium ion transmembrane transport, potassium ion transport, digestive tract development, neuropeptide signaling pathway, potassium ion import across plasma membrane, digestive tract morphogenesis, embryonic organ morphogenesis, regulation of peptide secretion, regulation of peptide transport in the stomach of 5-month-old embryo (Figure 3C). Additionally, KEGG analysis identified gastric acid secretion as a critical signaling pathway among the upregulated genes (Figure 3D). When compared to the stomach of 4-month-old embryo, heatmaps demonstrated a significant increase in the expression of genes associated with gastric acid secretion, including *KCNQ1*, *ATP4A*, *HRH2*, *KCNJ16*, *KCNE2*, *SLC4A2*, and *ATP4B* in the stomach of 5-month-old embryo (Figure 3E).

### Analysis of high-expression genes and biological processes in the stomach of 6-month-old human embryo

**FIGURE 4:**
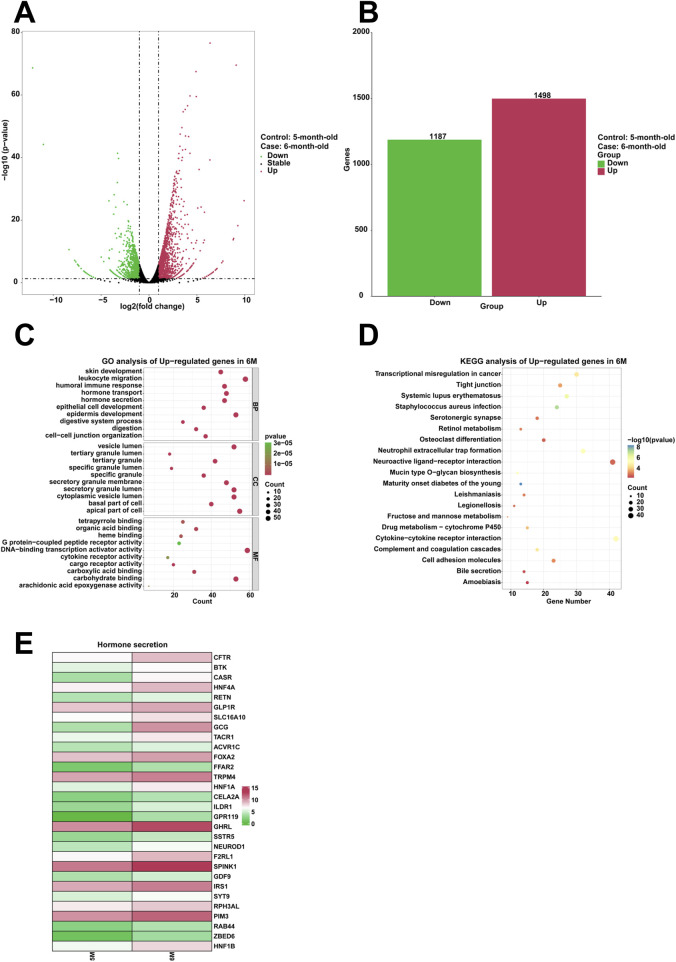
Analysis of the highly expressed genes and biological processes in the stomach of 6-month-old human embryo. **(A)** Volcano plot illustrating the differentially expressed genes (DEGs) between the stomach tissues of 5-month-old and 6-month-old embryos. **(B)** Statistical analysis of 1,498 upregulated and 1,187 downregulated genes in the stomach of 6-month-old embryo. **(C)** GO analysis of the upregulated genes in the stomach tissue of 6-month-old embryo. **(D)** KEGG pathway analysis of the upregulated genes identified in the stomach of 6-month-old embryo. **(E)** Heatmap revealed the expression genes related to hormone secretion in the stomachs of 5-month-old and 6-month-old embryos.

To examine highly expressed genes and their associated biological functions in the stomach of 6-month-old human embryo, RNA sequencing was employed to identify differentially expressed genes (DEGs) between the stomachs of 5-month-old and 6-month-old embryos. The analysis indicated that 1,498 genes were upregulated, while 1,187 genes were downregulated in the 6-month-old stomach (Figures 4A,B). GO analysis revealed the enrichment of processes related to humoral immune response, hormone transport, hormone secretion, epithelial cell development, epidermis development, digestion within the 6-month-old stomach (Figure 4C). KEGG analysis demonstrated that serotonergic synapse was significantly enriched among the upregulated genes (Figure 4D). Compared to the 5-month-old stomach, heatmaps illustrated a notable increase in the expression of genes associated with hormone secretion in the 6-month-old stomach, including *CFTR*, *BTK*, *CASR*, *HNF4A*, *RETN*, *GLP1R*, *SLC16A10*, *GCG*, *TACR1*, *ACVR1C*, *FOXA2*, *FFAR2*, *TRPM4*, *HNF1A*, *CELA2A*, *ILDR1*, *GPR119*, *GHRL*, *SSTR5*, *NEUROD1*, *F2RL1*, *SPINK1*, *GDF9*, *IRS1*, *SYT9*, *RPH3AL*, *PIM3*, *RAB44*, *ZBED6*, and *HNF1B* (Figure 4E).

### Analysis of high-expression genes and biological processes in the stomach of 7-month-old human embryo

**FIGURE 5:**
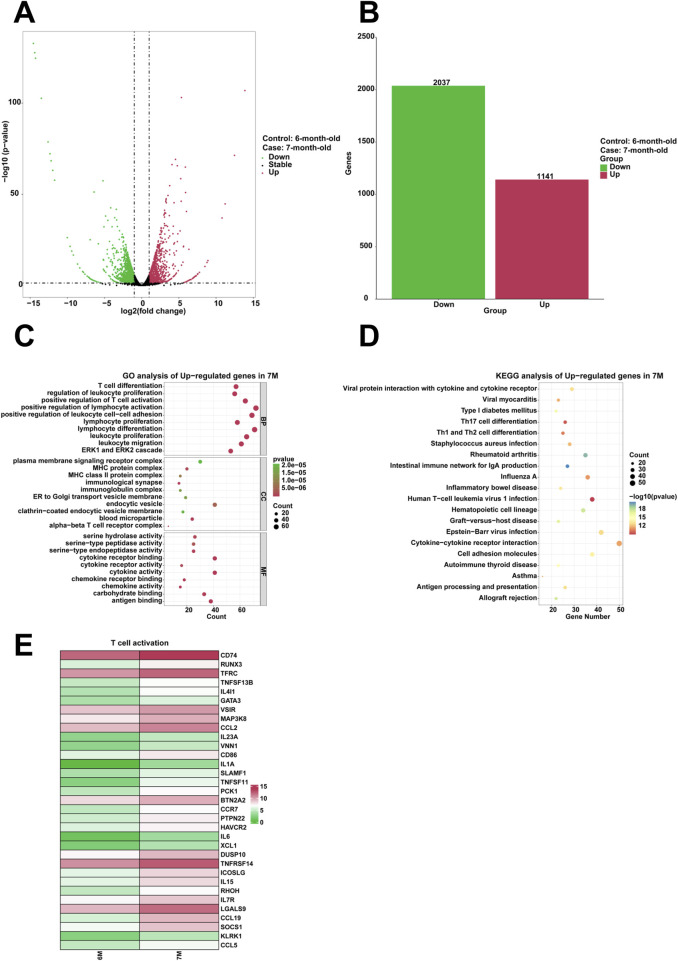
Analysis of the highly expressed genes and biological processes in the stomach of 7-month-old human embryo. **(A)** Volcano plot illustrating the differentially expressed genes (DEGs) between the stomach tissues of 6-month-old and 7-month-old embryos. **(B)** Statistical analysis of 1,141 upregulated and 2,037 downregulated in the stomach of 7-month-old embryo. **(C)** GO analysis of the upregulated genes in the stomach tissue of 7-month-old embryo. **(D)** KEGG pathway analysis of the upregulated genes identified in the stomach of 7-month-old embryo. **(E)** Heatmap revealed the expression genes related to T cell activation in the stomachs of 6-month-old and 7-month-old embryos.

To examine the genes that are highly expressed and their corresponding biological functions in the stomach of 7-month-old human embryo, RNA sequencing analysis was conducted to identify differentially expressed genes (DEGs) between the stomachs of 6-month-old and 7-month-old embryos. A comparison revealed that 1,141 genes were upregulated and 2,037 genes were downregulated in the stomach of 7-month-old embryo (Figures 5A,B). To investigate the biological functions of differentially expressed genes (DEGs), GO analysis indicated the enrichment of processes associated with T cell differentiation, regulation of leukocyte proliferation, positive regulation of T cell activation, positive regulation of lymphocyte activation, positive regulation of leukocyte cell-cell adhesion, leukocyte proliferation, lymphocyte differentiation, lymphocyte proliferation, leukocyte migration in the stomach of 7-month-old embryo (Figure 5C). Furthermore, KEGG analysis identified human T-cell leukemia virus 1 infection a critical signaling pathway among the upregulated genes (Figure 5D). When compared to the stomach of 6-month-old embryo, heatmaps demonstrated a significant increase in the expression of genes associated with positive regulation of T cell activation, including *CD74*, *RUNX3*, *TFRC*, *TNFSF13B*, *IL4I1*, *GATA3*, *VSIR*, *MAP3K8*, *CCL2*, *IL23A*, *VNN1*, *CD86*, *IL1A*, *SLAMF1*, *TNFSF11*, *PCK1*, *BTN2A2*, *CCR7*, *PTPN22*, *HAVCR2*, *IL6*, *XCL1*, *DUSP10*, *TNFRSF14*, *ICOSLG*, *IL15*, *RHOH*, *IL7R*, *LGALS9*, *CCL19*, *SOCS1*, *KLRK1*, and *CCL5* in the stomach of 7-month-old embryo (Figure 5E).

### Analysis of gene expression profiles and associated biological processes in the stomach across the pseudotime from 3-month-old to 7-month-old

**FIGURE 6:**
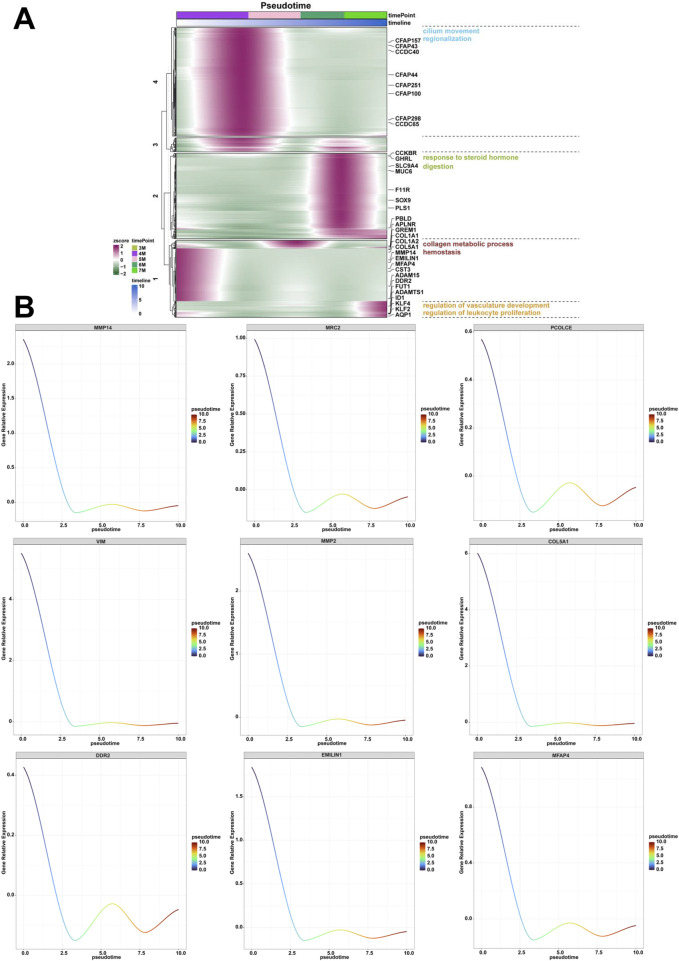
Analysis of the genes and biological processes along the pseudotime from the 3-month-old to 7-month-old stomach. **(A)** The biological processes of collagen metabolic process and hemostasis, cilium movement and regionalization, response to steroid hormone and digestion, regulation of vasculature development and regulation of leukocyte proliferation along the pseudotime from the 3-month-old to 7-month-old stomach. **(B)** The expression of *MMP14*, *MRC2*, *PCOLCE*, *VIM*, *MMP2*, *COL5A1*, *DDR2*, *EMILIN1*, *MFAP4* along the pseudotime from the 3-month-old to 7-month-old embryonic stomach.

To investigate the dynamic expression profiles, evolutionary conservation patterns, and functional networks of genes associated with human embryonic stomach ranging from 3-month-old to 7-month-old, we assigned the genes with time series characteristics, analyzed the temporal trends of gene expression and subsequent categorization into distinct clusters based on expression patterns. Through GO and KEGG analysis, we identified multiple biological processes with temporal characteristics during the embryonic development of stomach. Genes with similar expression patterns along the pseudotime of 4-month-old stomach exhibited enrichment in biological processes related to collagen metabolism and hemostasis (Figure 6A). The identified genes included *MMP14*, *MRC2*, *PCOLCE*, *VIM*, *MMP2*, *COL5A1*, *DDR2*, *EMILIN1*, *MFAP4* (Figure 6B). Similarly, genes with similar expression patterns along the pseudotime from 4-month-old to 5-month-old stomach exhibited enrichment in biological processes of cilium movement and regionalization (Figure 6A). The identified genes included *CFAP100*, *DNAAF1*, *CLXN*, *CCDC40*, *CCDC65*, *CFAP157* (Supplementary Figure S1A). Genes with similar expression patterns along the pseudotime from 6-month-old to 7-month-old stomach exhibited enrichment in biological processes related to response to steroid hormone and digestion (Figure 6A). The expression of genes such as *MUC6*, *GHRL*, *F11R*, *PLS1*, *VSIG1*, *SST*, *SPINK1* and *SOX9* was identified (Supplementary Figures S1B, S2A). Genes with similar expression patterns along the pseudotime of 7-month-old stomach exhibited enrichment in biological processes related to regulation of vasculature development and regulation of leukocyte proliferation (Figure 6A). The identified genes included *WARS1*, *THBS1*, *STAT1*, *RHOB*, *FUT1*, *ADAMTS1*, *ID1* and *AQP1* was identified (Supplementary Figure S2B).

### Construction of regulatory networks and verification of genes associated with embryonic digestive tract development, digestion and embryonic organ morphogenesis

**FIGURE 7:**
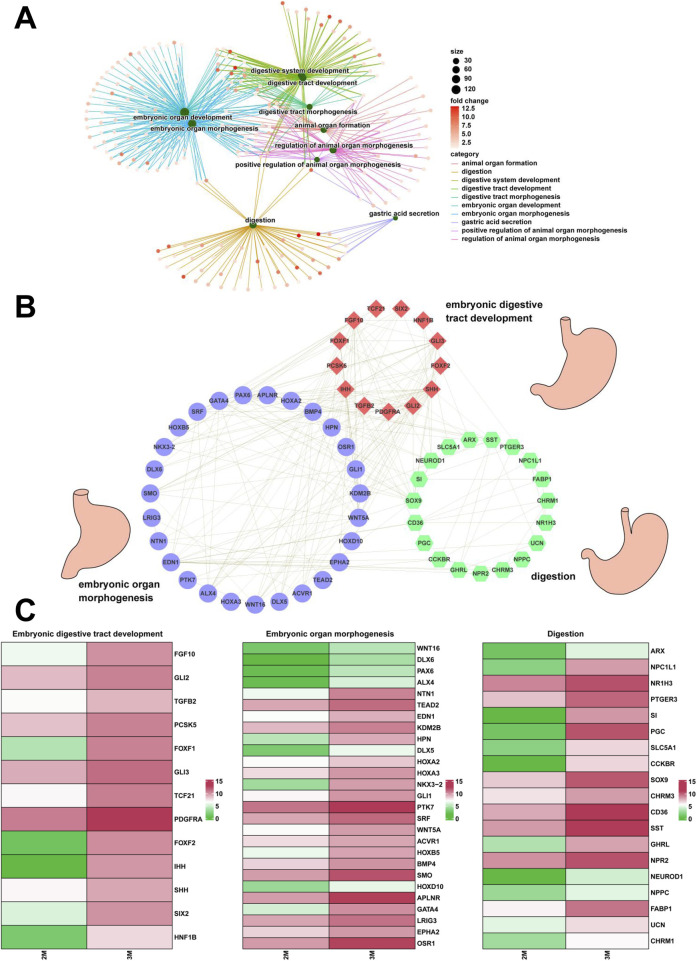
The regulatory network associated with stomach development. **(A)** The interaction networks associated with 10 biological processes of the upregulated genes in the stomach of 3-month-old embryo. **(B)** The interaction networks of genes associated with embryonic digestive tract development, digestion and embryonic organ morphogenesis. **(C)** The heatmap showed the expression of genes associated with embryonic digestive tract development, digestion and embryonic organ morphogenesis between the 2-month-old and 3-month-old stomachs.

**FIGURE 8:**
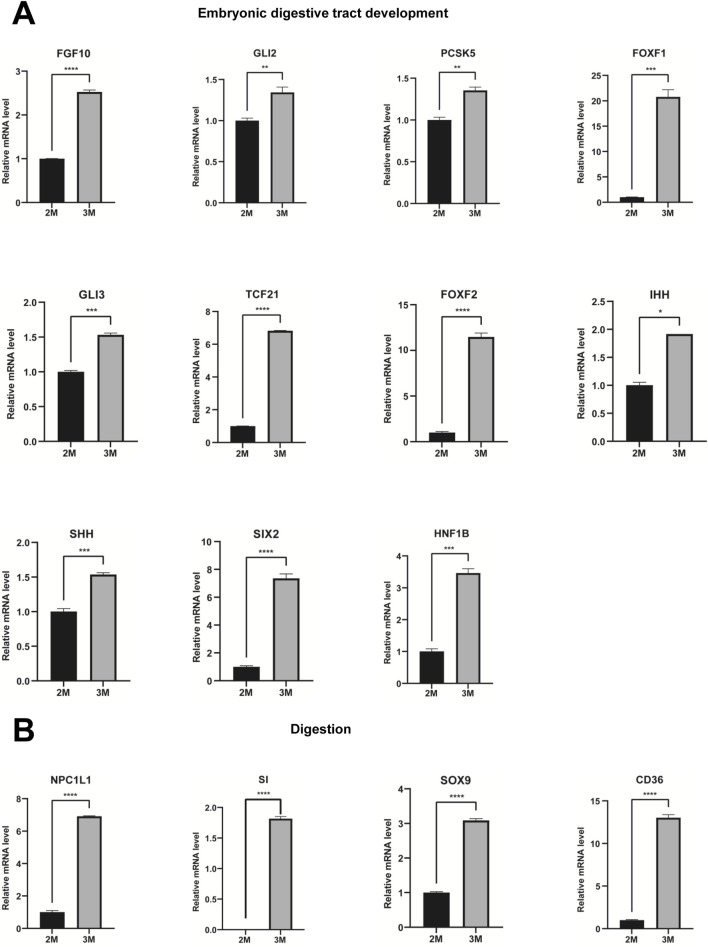
Verification of genes associated with embryonic digestive tract development and digestion. **(A)** The expression of genes associated with embryonic digestive tract development in the stomachs of 2-month-old and 3-month-old human embryos using qPCR. **(B)** The expression of genes involved in digestion in the stomachs of 2-month-old and 3-month-old human embryos using qPCR (*P < 0.05, **P < 0.01, ***P < 0.001 and ****P < 0.0001).

**FIGURE 9:**
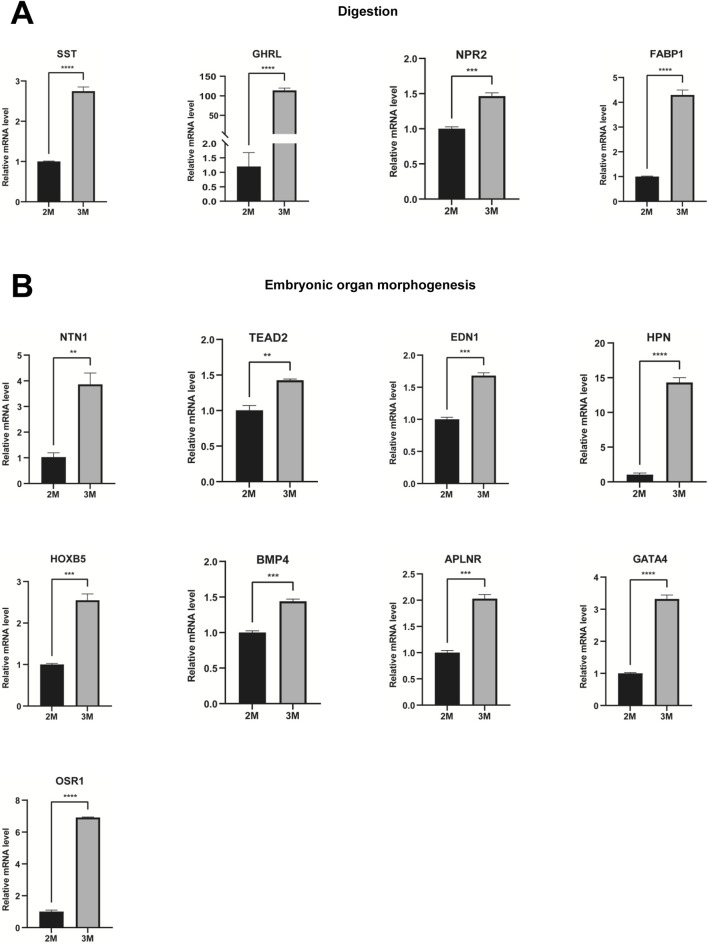
Verification of genes associated with digestion and embryonic organ morphogenesis. **(A)** The expression of genes associated with digestion in the stomachs of 2-month-old and 3-month-old human embryos using qPCR. **(B)** The expression of genes involved in embryonic organ morphogenesis in the stomachs of 2-month-old and 3-month-old human embryos using qPCR (*P < 0.05, **P < 0.01, ***P < 0.001 and ****P < 0.0001).

Through comparative transcriptome sequencing analysis of stomachs from 2-month-old and 3-month-old human specimens, we illustrated the regulatory network involved in 10 biological processes of upregulated genes in the stomach of 3-month-old embryo (Figure 7A). Additionally, we also identified highly expressed genes that are associated with embryonic digestive tract development, digestion and embryonic organ morphogenesis. The network of genes involved in these processes is illustrated Figure 7B. The expression levels of genes implicated in embryonic digestive tract development, digestion and embryonic organ morphogenesis were significantly upregulated in the 3-month-old stomach compared to the 2-month-old counterpart. Key genes include *FGF10*, *GLI2*, *TGFB2*, *PCSK5*, *FOXF1*, *GLI3*, *TCF21*, *PDGFRA*, *FOXF2*, *IHH*, *SHH*, *SIX2*, *HNF1B*, *PAX6*, *PTK7*, *DLX6*, *OSR1*, *WNT16*, *TEAD2*, *HOXA2*, *EPHA2*, *SMO*, *HOXA3*, *KDM2B*, *HPN*, *GATA4*, *HOXB5*, *EDN1*, *HOXD10*, *APLNR*, *ACVR1*, *NKX3-2*, *WNT5A*, GLI1, *BMP4*, *ALX4*, *LRIG3*, *SRF*, *NTN1*, *DLX5*, *SI*, *CHRM3*, *NEUROD1*, *PTGER3*, *SST*, *CCKBR*, *SOX9*, *NR1H3*, *PGC*, *GHRL*, *ARX*, *NPR2*, *CD36*, *NPPC*, *NPC1L1*, *SLC5A1*, *FABP1*, *UCN*, and *CHRM1* (Figure 7C). Quantitative PCR (qPCR) analysis corroborated the expression of these genes, yielding results that are consistent with RNA sequencing (Figures 8, 9).

### Lowly expressed genes and biological processes in stomachs at various developmental stages

To investigate downregulated expressed genes and associated biological processes in stomachs at various developmental stages, we identified downregulated genes in these stages using RNA sequencing analysis. Compared to the 2-month-old stomach, GO analysis revealed that downregulated genes were significantly enriched in steroid metabolic process and steroid biosynthetic process in the 3-month-old stomach (Supplementary Figure S3A). KEGG analysis indicated that the steroid biosynthesis signaling pathway was represented among the downregulated genes in the 3-month-old stomach (Supplementary Figure S3B). Compared to the 3-month-old stomach, GO analysis revealed that downregulated genes were notably enriched in embryonic organ morphogenesis, regionalization, pattern specification process in the 4-month-old stomach (Supplementary Figure S3C). KEGG analysis revealed that the protein digestion and absorption signaling pathway was represented among the downregulated genes in the 4-month-old stomach (Supplementary Figure S3D). In the 5-month-old stomach, GO analysis showed that downregulated genes were notably enriched in cilium assembly and cilium organization (Supplementary Figure S3E). KEGG analysis indicated that the tyrosine metabolism signaling pathway was represented among the downregulated genes in the 5-month-old stomach (Supplementary Figure S3F). In the 6-month-old stomach, GO analysis revealed that downregulated genes were significantly enriched in the regulation of synaptic plasticity and regulation of neurotransmitter levels (Supplementary Figure S3G). KEGG analysis revealed that the neuroactive ligand-receptor interaction signaling pathway was represented among the downregulated genes in the 6-month-old stomach (Supplementary Figure S3H). In the 7-month-old stomach, GO analysis revealed that downregulated genes were notably enriched in embryonic skeletal system development and chromosome segregation (Supplementary Figure S3I). KEGG analysis revealed that the neuroactive ligand-receptor interaction and neutrophil extracellular trap formation signaling pathways were represented among the downregulated genes in the 7-month-old stomach (Supplementary Figure S3J).

## Discussion

During embryogenesis, the proper development of the human embryonic stomach is crucial for maintaining homeostasis and preventing disease in the adult stomach. In this study, we collected embryonic stomachs ranging from two to 7 months of age. To investigate the genes that are highly expressed and associated biological processes occurring in different stages of stomach development, we employed RNA sequencing analysis to identify differentially expressed genes (DEGs). We identified genes and biological processes associated with embryonic digestive tract development, digestion, and organ morphogenesis. The results provide essential insights for research into the development and functionality of embryonic stomach.

Compared to the 2-month-old stomach, the development of digestive system is evident in the upregulated genes of the 3-month-old stomach (Figure 1C). Genes such as *FGF10*, *SOX9*, *GATA4*, *SFRP2*, *BARX1*, *PDX1*, *KIT*, *NPR2*, *IHH*, *SHH*, *CDX2*, *SIX2*, *FOXL1*, *EPHB3*, *HNF1B*, *CLDN18*, and others are markedly enriched in the 3-month-old stomach (Figure 1E). Previous studies have demonstrated that FGF10 plays a pivotal role in maintaining stomach progenitors, morphogenesis, and cellular differentiation (Nyeng et al., 2007). SOX9 regulates the transformation of gastric stem cells by influencing asymmetric cell division (Chen et al., 2023). GATA4 orchestrates epithelial morphogenesis in the developing stomach, facilitating the formation of glandular columnar epithelium (DeLaForest et al., 2021). During gut organogenesis, BARX1 expression is restricted to the mesenchyme of stomach (Kim et al., 2005). BARX1 orchestrates the expression of two secreted WNT antagonists in mesenchymal cells, sFRP1 and sFRP2, which functionally compensate for the role of BARX1 (Kim et al., 2005). KIT signaling is essential for developing coordinated motility patterns in the zebrafish gastrointestinal tract (Rich et al., 2013). FOXF1 and FOXL1 mediate hedgehog signaling and regulate epithelial proliferation in the developing stomach and intestine (Madison et al., 2009). The elevated expression levels of these genes provide key insights into the developmental processes in the 3-month-old embryo, indicating that the formation and maturation of the digestive system are vital during this stage. This period of embryonic development is crucial as it lays the foundational structures for proper nutrient absorption and metabolism necessary for further growth and development. In clinical and applied research, genes *FGF10*, *SOX9*, *GATA4*, *BARX1*, *PDX1* and *KIT*, which exhibit upregulated expression patterns during stomach development, play pivotal roles in stomach morphogenesis and epithelial differentiation. Dysregulation of these molecules is mechanistically linked to developmental disorders such as gastric hypoplasia. These genes may serve as potential biomarkers for monitoring normal stomach developmental patterning.

In addition, the upregulated genes in 3-month-old stomach are also predominantly associated with critical developmental processes, including axon guidance, neuron projection guidance, mesenchymal cell differentiation, epithelial to mesenchymal transition. Genes such as *PAX6*, *SEMA3A*, *SEMA3B*, *NTN1*, *IGSF9*, *FLRT3*, *CHL1*, and others are significant enrichment in the 3-month-old stomach (Figure 1F). Extensive evidence has demonstrated that the transcription factor PAX6 coordinately regulates both axon guidance and fasciculation processes in developing retinal ganglion cells during retinogenesis (Lalitha et al., 2020). Semaphorin-3A (SEMA3A) is a guidance protein that plays a crucial role in the signaling pathways involved in the collapse of growth cones in the nervous system (Kim et al., 2023). Semaphorin-3B (SEMA3B), a secreted axon guidance protein predominantly localized in neurons, critically regulates activity-dependent synaptic plasticity (Du et al., 2022). Netrin-1, encoded by the NTN1 gene, is an axon guidance protein that critically regulates cell survival and tumorigenesis (Nakayama et al., 2022). IGSF9 family members play pivotal roles in regulating neurite outgrowth and branching, axon guidance, synaptic maturation (Hansen and Walmod, 2013). FLRT3 interacting with Robo1 protein regulates the chemoattraction of Netrin-1 during neural development (Leyva-Diaz et al., 2014). Recently, studies have reported that sensory nerves promote the growth and metastasis of gastric cancer through the signaling pathway formed by calcitonin gene-related peptide (CGRP) and its receptors (Zhi et al., 2025). High expression profiling of these genes reveals potential synergistic regulatory mechanisms between neuronal network formation and embryonic gastric morphogenesis during early embryogenesis. Biological process analysis demonstrates that axon guidance molecules not only participate in neural development, but also regulate critical biological processes including mesenchymal cell differentiation and epithelial-mesenchymal transition, thereby contributing to gastric development and functional maturation. Although this study provides initial insights into the potential role of axon guidance pathways in embryonic gastric development, further validation through organoid models and conditional gene knockout technology is required to elucidate the specific regulatory mechanisms of key signaling molecules in gastric wall stratification and neural crest cell migration.

Compared to the stomach of 3-month-old embryo, the 4-month-old stomach exhibits biological processes associated with various aspects of ciliary function, including cilium movement, cilium organization, cilium assembly, cilium or flagellum-dependent cell motility, cilium-dependent cell motility, cilium movement involved in cell motility (Figure 2C). Genes such as *ZMYND10*, *WDR54*, *SPAG6*, *RABL2B*, *RFX3*, *DNAAF6*, *IFT25*, *CFAP61*, *TEKT2*, *SPEF1*, *CCDC113*, *KLC3*, *ODAD1*, *CFAP69*, *IFT56*, *CEP126*, *RSPH4A*, *NME5*, *IFT57*, *IQCG*, *ROPN1B*, *DNAH1*, *ARMC2*, *DNAH7*, *BBOF1*, *DAW1*, *TEKT3*, and *DNAAF11* exhibited significant enrichment in the stomach of 4-month-old human embryo (Figure 2E). ZMYND10 plays a pivotal role in maintaining the stability of intermediate chain proteins during the cytoplasmic pre-assembly process of dynein arms (Cho et al., 2018). SPAG6, integral to the axonemal central apparatus, is crucial for the functionality of cilia in ependymal cells and flagella in sperm (Teves et al., 2014). RFX3 functions as a transcriptional co-activator to FOXJ1, facilitating the induction of ciliary gene expression during the differentiation of basal/progenitor cells into ciliated cells (Didon et al., 2013). The interaction between LARP6 and DNAAF6 within biomolecular condensates is critical for ciliogenesis in multiciliated cells (Earwood et al., 2024). Biallelic mutations in CFAP61 lead to morphological irregularities in flagella, resulting in male infertility (Ma et al., 2021). The microtubule-bundling protein SPEF1 facilitates the formation of the central apparatus in mammalian cilia (Zheng et al., 2019). The CCDC113/CCDC96 complex mediates signal transmission from RS3 and the N-DRC to dynein subunit g, thereby regulating its activity and modulating the pattern of ciliary beating (Bazan et al., 2021). Ciliogenesis-associated kinase 1 (CILK1), essential for ciliary formation, localizes within primary cilia and governs intra-ciliary transport (Rah et al., 2022). IFT56 is pivotal in the regulation of vertebrate developmental patterning, as it maintains the integrity of the IFT-B complex and the architecture of ciliary microtubules (Xin et al., 2017). These processes, crucial for cell motility, were specifically identified in the 4-month-old stomach, suggesting a developmental progression in ciliary functions. Thus, as the embryo matures, there is a distinct progression in ciliary functions that are vital for its development. The expression patterns of these genes serve as molecular indicators of the intricate cellular activities that underpin the dynamic development of ciliary structures during this critical phase of embryogenesis.

Compared to the stomach of 4-month-old embryo, the 5-month-old stomach exhibited significant advancements in various biological processes, including digestive system development, digestive tract morphogenesis, embryonic organ morphogenesis, regulation of peptide secretion, regulation of peptide transport (Figure 3C). Notably, the pathway for gastric acid secretion was identified through the upregulated genes in the stomach of 5-month-old human embryo (Figure 3D). Genes implicated in the gastric acid secretion pathway, such as *KCNQ1*, *ATP4A*, *HRH2*, *KCNJ16*, *KCNE2*, *SLC4A2*, and *ATP4B*, exhibited significant upregulation in the stomach of 5-month-old human embryo (Figure 3E). KCNQ1, also known as Kv7.1, is a voltage-dependent potassium (K+) channel critical for the regulation of gastric acid secretion (Sun and MacKinnon, 2020). ATP4A is a pivotal tumor suppressor gene that encodes H^+^, K^+^-ATPase, which mediates gastric acid secretion in the stomach (Cao et al., 2020). The activation of histamine receptor 2 (H2) in the stomach stimulates gastric acid secretion. H2 receptor antagonists are employed in the treatment of peptic ulcers and acid reflux (Marquez-Gomez et al., 2022). In parietal cells, the apical potassium channels, comprising the KCNQ1 alpha subunit and the KCNE2 beta subunit, facilitate K^+^ efflux to promote gastric acid secretion via the H^+^K^+^-ATPase (Roepke et al., 2010). The SLC4A2 gene plays an integral role in gastric acid secretion, contributes to spermatogenesis, and is involved in osteoclastogenesis (Xue et al., 2023). The hydrogen/potassium ATPase β-subunit (ATP4B) acts as a crucial proton pump in gastric acid secretion (Pan et al., 2021). The elevated expression of these genes in 5-month-old specimens indicates significant development within the digestive system, particularly highlighting the maturation of gastric acid secretion pathways. The pronounced gene activity suggests that these pathways of gastric acid secretion play critical roles in the digestion and breakdown of food. In the field of clinical and applied research, key genes regulating gastric acid secretion (including KCNQ1, ATP4A, HRH2, KCNJ16, KCNE2, SLC4A2, and ATP4B) are upregulated in the 5-month-old stomach. The expression patterns of these genes show strong correlation with gastric acid secretory capacity. Mutations in these genes may be linked to congenital chloride diarrhea and achlorhydria. These genes may serve as promising biomarkers for assessing developmental competence in gastric acid production.

In comparison to the stomach of 5-month-old, the 6-month-old stomach exhibits enhanced biological processes, including humoral immune response, hormone transport, hormone secretion, epithelial cell development, epidermis development, digestion (Figure 4C). The genes implicated in hormone secretion, including *CFTR*, *BTK*, *CASR*, *HNF4A*, *RETN*, *GLP1R*, *SLC16A10*, *GCG*, *TACR1*, *ACVR1C*, *FOXA2*, *FFAR2*, *TRPM4*, *HNF1A*, *CELA2A*, *ILDR1*, *GPR119*, *GHRL*, *SSTR5*, *NEUROD1*, *F2RL1*, *SPINK1*, *GDF9*, *IRS1*, *SYT9*, *RPH3AL*, *PIM3*, *RAB44*, *ZBED6*, and *HNF1B*, were significantly upregulated in the stomach of 6-month-old specimens (Figure 4E). The stomach functions as a critical endocrine organ by producing various peptide hormones essential for enteric and systemic physiological processes, such as ghrelin and leptin (O'Connor and O'Morain, 2014). The calcium-sensing receptor (CaSR) plays a pivotal role in regulating parathyroid hormone secretion and renal calcium reabsorption (Kettritz, 2020). Resistin (RETN), a hormone secreted by adipocytes, significantly regulates glucose and lipid metabolism (Zhang et al., 2021). The SLC16A10 gene encodes MCT10, a transmembrane transporter responsible for the cellular uptake of thyroid hormones (Girgis et al., 2021). The Gcg gene is responsible for encoding several peptides, including glucagon, glucagon-like peptide-1, glucagon-like peptide-2, oxyntomodulin, and glicentin (Sandoval and D'Alessio, 2015). Glucagon-like peptide 1 (GLP-1), a 30-amino acid peptide hormone, is synthesized by intestinal epithelial endocrine L-cells through the differential processing of proglucagon (Holst, 2007). ILDR1 has been reported to function as a lipoprotein receptor that mediates the secretion of the fat-induced cholecystokinin (CCK) hormone in the small intestine (Morozko et al., 2015). The high expression of these genes suggests the hormone secretion and digestion are the main biological processes in the 6-month-old stomach. Specifically, it is observed that the regulatory mechanisms governing peptide secretion are highly active, ensuring efficient enzymatic and hormonal release critical for digestive processes.

Compared to the stomach of 6-month-old human embryo, the 7-month-old stomach showed the biological processes, including T cell differentiation, regulation of leukocyte proliferation, positive regulation of T cell activation, positive regulation of lymphocyte activation, positive regulation of leukocyte cell-cell adhesion, leukocyte proliferation, lymphocyte differentiation, lymphocyte proliferation, leukocyte migration (Figure 5C). T-cell-mediated immunoregulation plays a pivotal role in maintaining homeostasis within the gastrointestinal tract (Saurer and Mueller, 2009). T-cell cytokines significantly affect epithelial cell responses during *Helicobacter pylori* infection (Algood, 2020). Innate lymphoid cells (ILCs) are particularly abundant in the gastrointestinal tract, where they interact with commensal bacteria, pathogens, and other local microenvironmental elements. These interactions are critical in modulating the host’s immune responses to infections and oncogenic processes (Jiao et al., 2023). Regulating lymphocyte proliferation could be a key mechanism that contributes to the development of gastric pathologies (Ferrand et al., 2008). The pronounced enrichment of specific biological processes suggests that immune responses are predominantly active in the stomach of 7-month-old embryo. This enrichment highlights the immune system’s critical role in influencing gastric activities at this developmental stage. Immune-related genes (CD74, GATA3, IL6, CCL5) exhibit upregulated transcriptional activity in clinical and translational research contexts. Aberrant immune activation during this stage may predispose to postnatal inflammatory gastric disorders. The combined elevation of these genes could serve as a predictive biomarker panel for identifying individuals at higher risk of developing postnatal inflammatory gastric disorders through non-invasive prenatal screening or early postnatal immune profiling. Furthermore, it is essential to further investigate how these immune processes shape the physiological state of the stomach during early life stages. Finally, we identified key biological processes including collagen metabolic process and hemostasis, cilium movement and regionalization, response to steroid hormone and digestion, regulation of vasculature development and regulation of leukocyte proliferation along the pseudotime from the 3-month-old to 7-month-old stomach (Figure 6A). These findings are consistent with previous observations, emphasizing the presence of distinct biological processes in the stomach during different developmental phases.

The literatures indicate that the glands and primary artery of the gastric fundus form during stage 6 (9–12 weeks of embryonic development), while the body of the stomach progressively develops in human embryo. This study yielded comparable results. Compared to the 2-month-old stomach, GO analysis revealed significant enrichment in numerous processes in the 3-month-old stomach, including digestive tract development, embryonic organ morphogenesis, embryonic digestive tract development, digestion, digestive system development, mesenchymal cell differentiation, neuron projection guidance, digestive tract morphogenesis, epithelial to mesenchymal transition, axon guidance (Figure 1C). Furthermore, KEGG pathway analysis revealed that the signaling pathways associated with protein digestion and absorption were significantly represented among the upregulated genes (Figure 1D). These findings indicate that both morphological and functional development of the stomach occur in the early stages of 3-month-old human embryo. Building upon these findings, Figure 7B illustrates the network of gene interactions relevant to embryonic digestive tract development, digestion and embryonic organ morphogenesis. The complex regulatory relationships among these genes were identified. The network illustrates genes associated with stomach development and function, many of which have been extensively documented in previous research literatures, including FGF10 (Nyeng et al., 2007), SOX9 (Chen et al., 2023), GATA4 (DeLaForest et al., 2021), FOXF1 and FOXL1 (Madison et al., 2009). Additionally, we also identified many previously unknown interacting genes, thereby laying the groundwork for future research into the roles of these unknown genes in embryonic stomach development and function. Furthermore, the network reveals that the development of human embryonic stomach is not a singular process, but rather multiple interacting biological processes that collectively facilitate stomach development and function. The realization of these interacting biological processes primarily occurs through the co-expression of genes across various biological processes. These results suggest that the normal development and functional acquisition of the stomach necessitate the coordinated expression of multiple genes.

## Conclusion

In this study, we examined the gene expression patterns throughout different stages of stomach development, ranging from two to 7 months of age. During the early stages of development, genes that play a pivotal role in the morphological and functional development of the stomach exhibit elevated expression levels in the stomach of 3-month-old human embryo. As the stomach develops, genes involved in cilium assembly and organization, peptide and hormone secretion and transportation, and immune response show heightened expression levels in the stomach of 4-month-old to 7-month-old embryo. These findings suggest that the morphological and functional development of the stomach occur in the early stages of developmental processes. As the stomach develops, its additional functions are progressively acquired. Furthermore, we have identified many genes previously reported to be involved in stomach development, as well as numerous genes of previously unknown function. This study establishes a foundation for researching stomach development and diseases associated with fetal stomach abnormalities.

## Data Availability

All datasets generated for this study are included in the article/Supplementary Material, further inquiries can be directed to the corresponding authors.
