# Supplementary figures and images for: Study on gene expression in stomach at different developmental stages of human embryos
Source: Front Cell Dev Biol. 2025 May 30;13:1564789. doi: 10.3389/fcell.2025.1564789 (PMC12162658; doi:10.3389/fcell.2025.1564789)

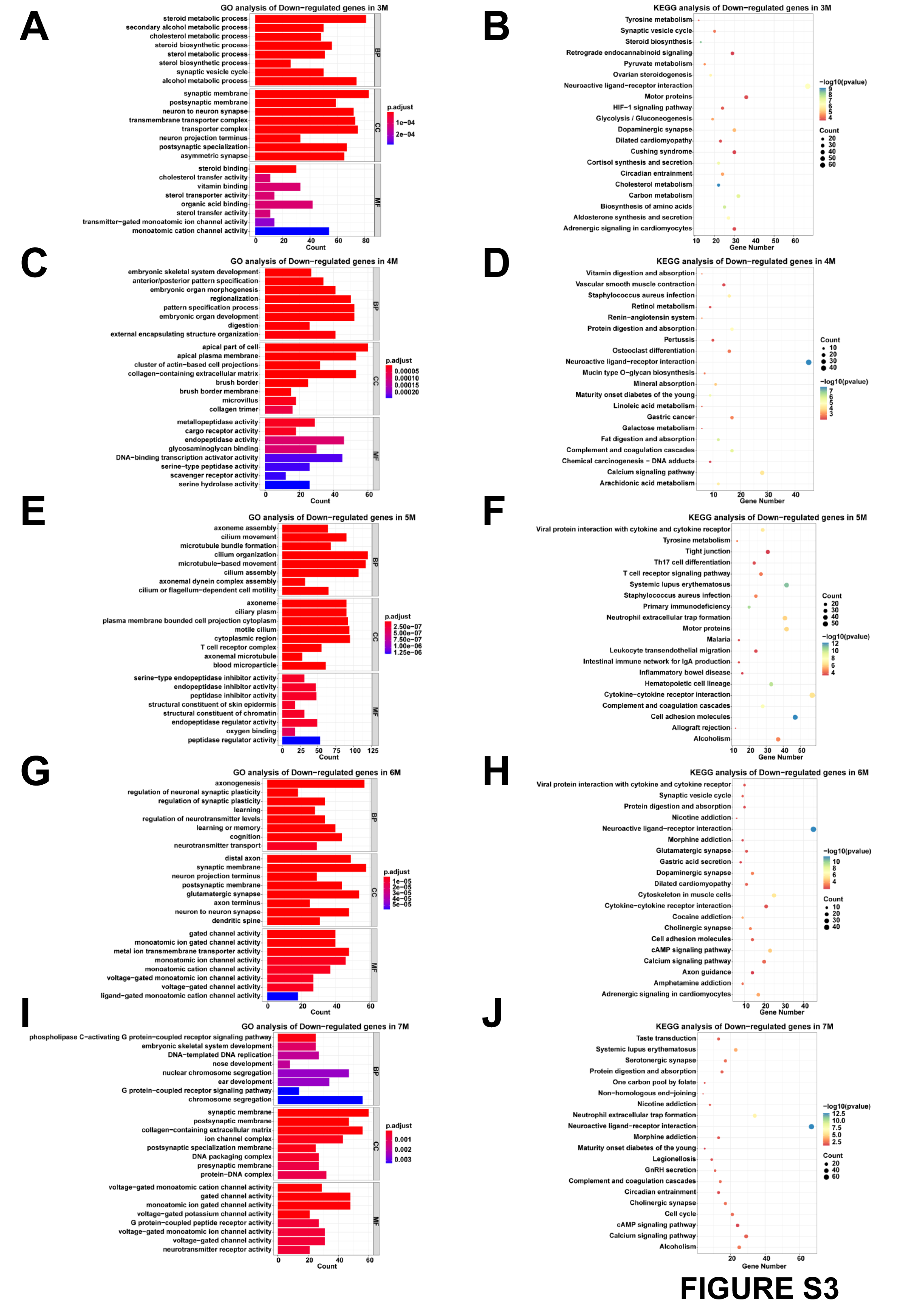

Supplement: Supplementary file 1 [file Image3.tif]

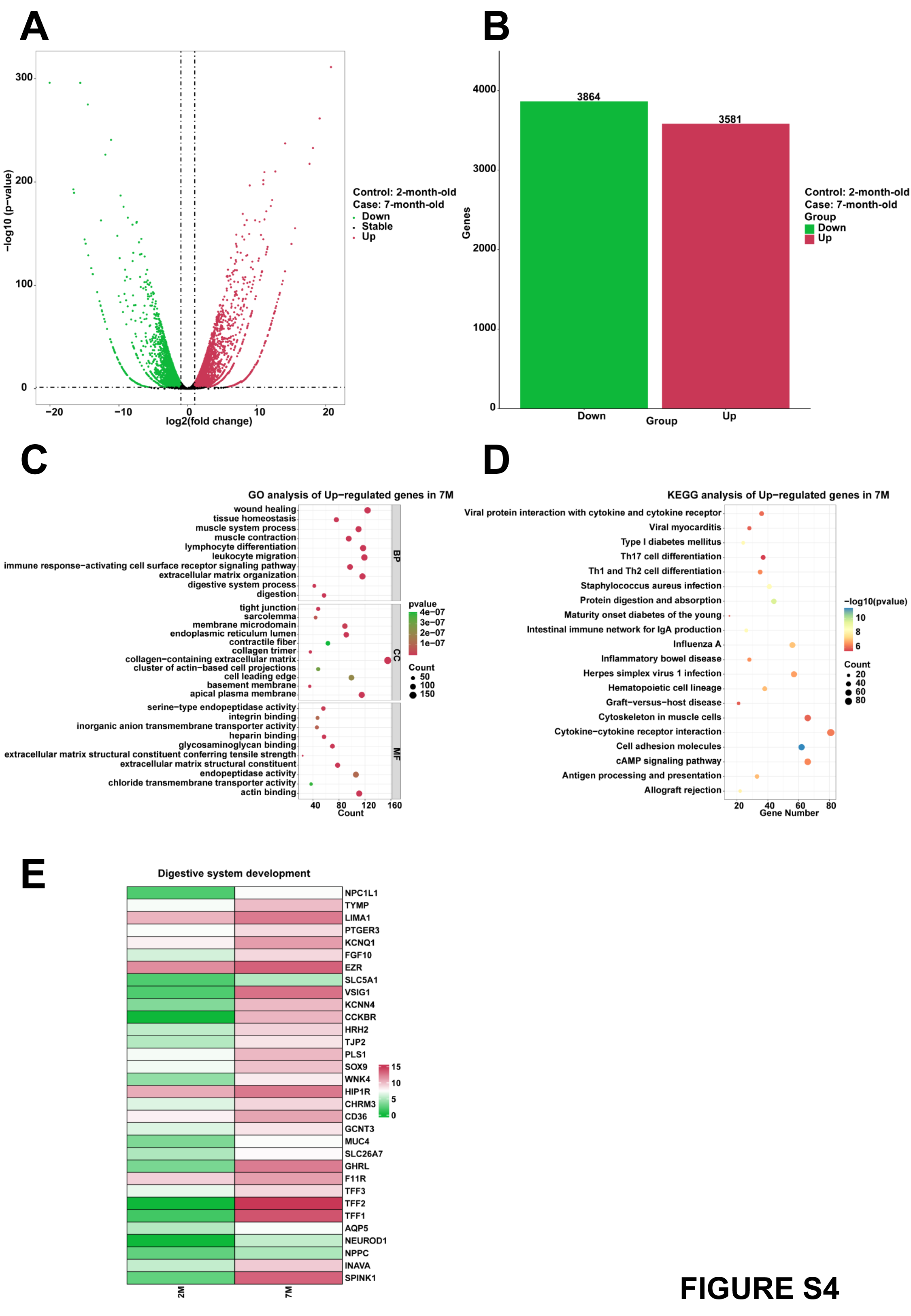

Supplement: Supplementary file 2 [file Image4.tif]

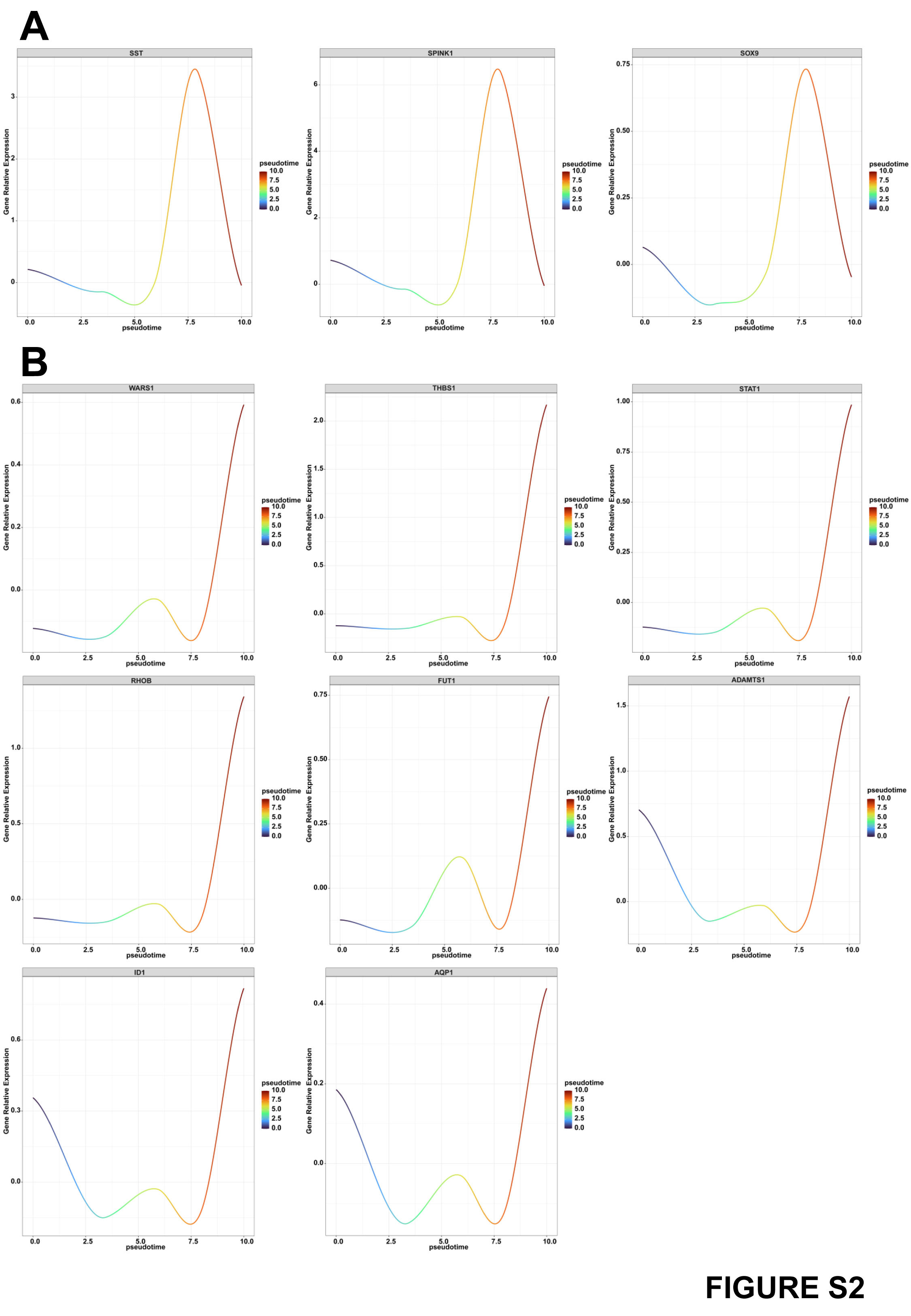

Supplement: Supplementary file 3 [file Image2.tif]

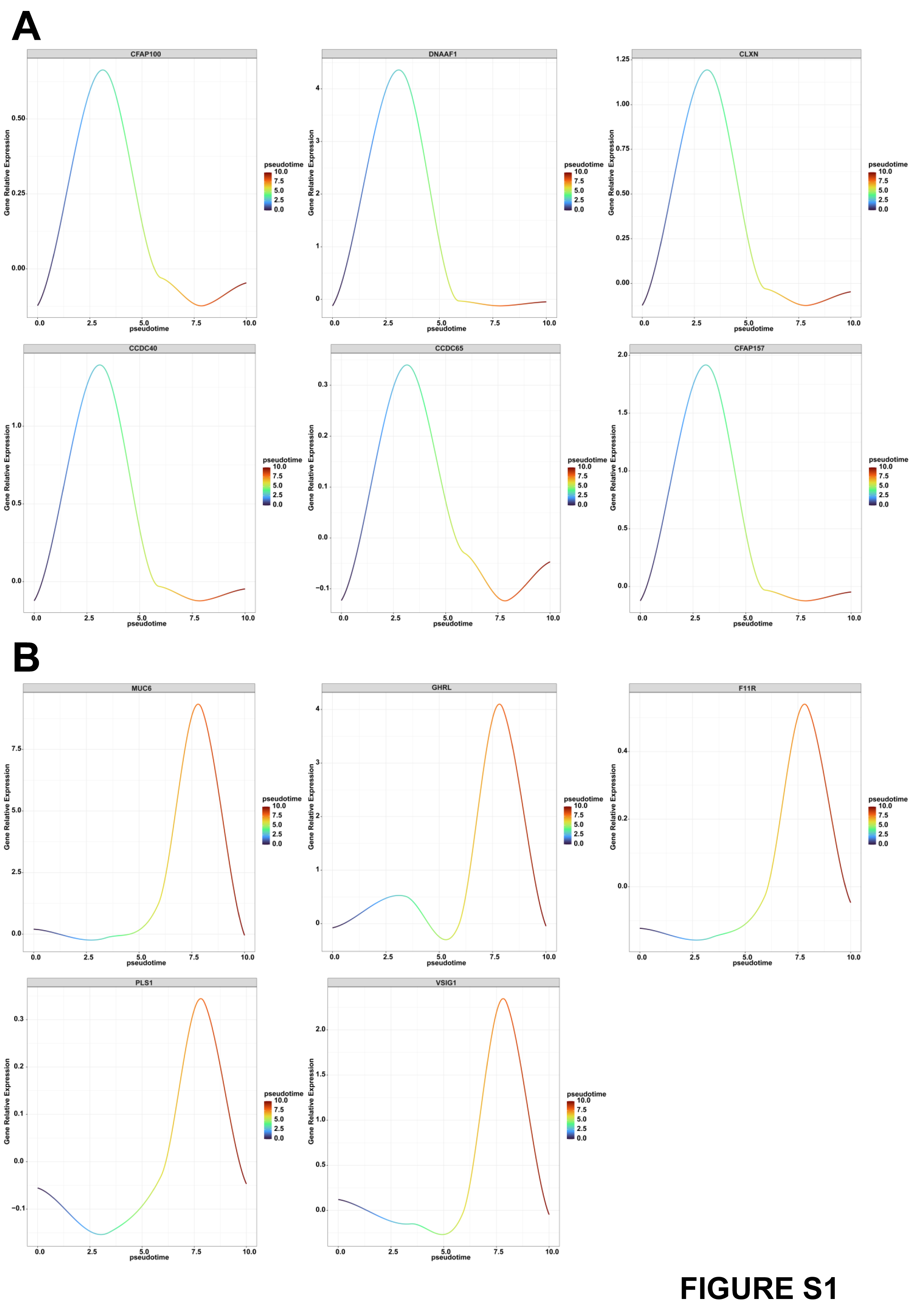

Supplement: Supplementary file 4 [file Image1.tif]

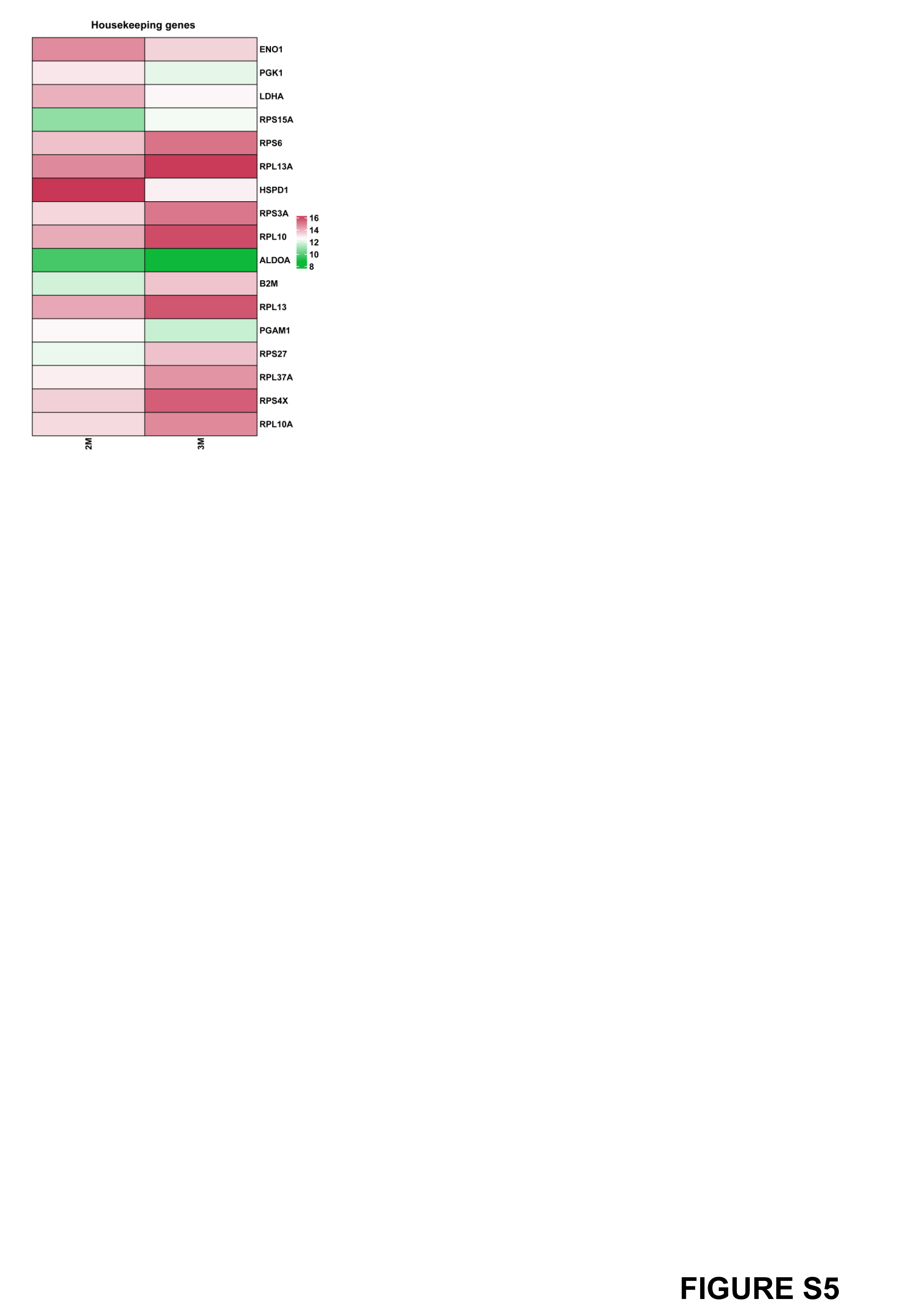

Supplement: Supplementary file 5 [file Image5.tif]
